# Exploring the role of community pharmacies as a harm reduction environment for anabolic–androgenic steroid consumers: triangulating the perspectives of consumers and pharmacists

**DOI:** 10.1186/s12954-024-00972-5

**Published:** 2024-03-13

**Authors:** Timothy Piatkowski, Sarah Benn, Lkhagvadulam Ayurzana, Michelle King, Sara McMillan, Laetitia Hattingh

**Affiliations:** 1 School of Applied Psychology, Griffith University, Gold Coast, QLD Australia; 2 Griffith Centre for Mental Health, Griffith University, Brisbane, QLD Australia; 3 School of Pharmacy and Medical Sciences, Griffith University, Gold Coast, QLD Australia; 4 Pharmacy Department, Gold Coast Health, Southport, QLD 4215 Australia

**Keywords:** Anabolic–androgenic steroids, Harm reduction, Injections, Pharmacies, Risk environment

## Abstract

**Background:**

While community pharmacies have been successful in providing harm reduction support for illicit substance consumers, little research has explored their role in addressing the needs of anabolic–androgenic steroid (AAS) consumers.

**Objective:**

This study aimed to triangulate the attitudes and experiences of AAS consumers and community pharmacist’s regarding AAS harm reduction.

**Methods:**

Semi-structured interviews were conducted with AAS consumers (*n* = 8) and community pharmacists (*n* = 15) between December 2022 and August 2023 in Australia. Interview data were analysed using reflexive thematic analysis.

**Results:**

While consumers emphasised easy access to pharmacies, particularly in urban areas, challenges were noted in rural regions. AAS consumers expressed a preference for community pharmacies, perceiving them as less confronting and a feasible avenue for accessing professional advice, highlighting the potential role of pharmacists in nurturing therapeutic alliances with AAS consumers. Similarly, pharmacists expressed receptivity to providing harm reduction information but acknowledged knowledge gaps, suggesting a need for tailored education programs to support AAS consumers effectively.

**Conclusions:**

Community pharmacies can be an important environment for AAS harm reduction. Strategies include utilising private spaces for open discussions with AAS consumers and enhancing pharmacists' understanding of AAS to foster trust and support. Further research is needed to address knowledge gaps and training needs for pharmacy staff, with the aim of creating a safer environment for AAS consumers.

**Supplementary Information:**

The online version contains supplementary material available at 10.1186/s12954-024-00972-5.

## Introduction

Community pharmacies are essential healthcare destinations that serve as an optimal resource for addressing non-urgent inquiries, such as safe injecting practices, management of adverse drug reactions, and medication provision, thus reducing the burden on general practitioners (GPs) [1, 2]. Pharmacists also have a role in addressing social determinants of health and promoting health equity, including the support of primary prevention strategies such as harm reduction interventions [3], for example through needle and syringe programs (NSPs) [4, 5]. In addition to offering advice and facilitating HIV/Hepatitis testing, pharmacists serve as a vital referral mechanism to various social, medical, and treatment services [6]. The experiences of pharmacists in this context have demonstrated predominantly positive outcomes associated with NSP provision [7, 8]. However, consumer attitudes to harm reduction service provision have been mixed [9], largely as a result of perceived systemic barriers for consumers which are often evident at sites of delivery [10, 11]. McVeigh et al. underscored the necessity for enhancing pharmacists' harm reduction training and implementing appropriate strategies to raise awareness of the needs of substance consumers, cater to the diverse needs of individuals who inject drugs, foster trusting relationships, and facilitate engagement within a confidential service setting [12].

One population of substance consumers that has often been overlooked in the literature in relation to community pharmacy access or use is anabolic–androgenic steroid (AAS) consumers. AASs are a group of performance and image enhancing drugs (PIEDs) which are increasingly used among non-athlete populations [13–17]. Investigations have demonstrated that AAS can enhance endurance, athletic performance and muscle growth, while reducing body fat [18, 19]. Existing literature on harm reduction in this area predominantly indicates that consumers may persist in AAS use due to perceived barriers to seeking help, concerns about muscle loss, and fears of worsening health upon cessation [20]. Despite increased interactions between frontline health workers and AAS users, the former often feel ill-equipped to address user needs [17, 21], leading users to seek support and advice from peers within non-judgmental ‘safe space [22, 23]. Considering these relationships between healthcare professionals and AAS consumers begs the question regarding whether there are environments which have yet to be considered for reducing harm among this unique cohort. While existing studies suggest that AAS consumers experience mistrust towards healthcare professionals due to perceived stigmatisation [23–25], it is unknown if this extends to community pharmacists.

### Theoretical framing

Risk environment frameworks suggest the convergence of various socio-structural factors that shape drug use behaviours and the associated risks [26]. Using this framework, Hanley Santos and Coomber [27] indicated that patterns of AAS use among consumers varied based on their motivations, prior knowledge, and experiences. Many users had limited knowledge about AAS before starting their use, relying on information from peers or suppliers, which was sometimes inaccurate or incomplete [27]. Therefore, they sought information from alternative sources, such as the internet and peer-using social networks [28–32]. When developing harm reduction interventions targeting AAS use, it is crucial to consider the broader context, including motivations and experiences [27], thus considering how we can ‘enable’ harm reduction to occur.

Consequently, Duff [33, 34] suggested that ‘enabling environments’ aim to contribute to the ongoing advancement of harm reduction frameworks. Advancement of frameworks involves outlining methodological propositions regarding the nature and organisation of enabling environments and the diverse social, material, and emotional resources available within them. Methodological analysis is crucial for understanding specific enabling environments, but Duff [35] emphasises that these environments are shaped by the practices, interactions, and behaviours of individuals and groups within them. Enabling environments are influenced by social, political, and economic processes, and focusing only on service provision overlooks the power of conduct and interaction in organising these places. By exploring the lived experiences of actors in these contexts, we recognise the diverse resources that contribute to the formation of such environments, particularly for substance consumers [26].

### The current study

Little research has explored the potential for community pharmacies within a risk environment framework, to act as sites of harm reduction for illicit substance use consumers [36–38], with no exploration of those who use AAS to our knowledge. In the context of enabling environments [39], community pharmacy services may offer a more comprehensive context to support AAS consumers in a way that reduces harm. Therefore, the aim of this study was to triangulate the attitudes and experiences of AAS consumers and community pharmacists toward AAS harm reduction within the context of the community pharmacy setting.

## Methods

### Design and ethics

This was an exploratory study was conducted according to the Consolidated Criteria for Reporting Qualitative Research [40] (see supplementary materials). University ethics committee approval was sought prior to study commencement (Approval Number: 2022/794).

### Sampling and recruitment

The study recruited 23 participants comprising two distinct samples: AAS consumers (*n* = 8) and community pharmacists (*n* = 15), each meeting specific inclusion criteria. Participants were identified through personal and professional networks and recruited via email and social media outreach, methods which have been documented successfully for both AAS consumers and healthcare providers [23]. Both groups were provided with study information in the form of a plain language statement. Consent was obtained verbally, and participants were assured of their right to withdraw. Upon completion, participants received a $40 gift card as appreciation for their time and expertise.

For the AAS consumer group, a total of eight participants (3 females, 5 males) aged 23 to 54 years (*Mean* = 35.5, SD = 8.6) were included. They reported diverse experiences with AAS, with the majority currently using injectable forms (6/8) and two using oral AAS. Differences were noted in the duration of use and the number of cycles they had undergone. On the other hand, the community pharmacist group comprised fifteen participants (8 females, 7 males) aged 25 to 49 years (*Mean* = 33.6, *SD* = 7.2). All participants were actively working in community pharmacies, with an average of 10 years of experience in this setting. Most were based in Queensland (*n* = 13), with two from New South Wales, and three working in regional areas. Among them, five were employed in independent pharmacies, while the rest worked in franchise pharmacies. The mean interview duration for this group was 36.4 min (*SD* = 12.6).

### Materials and data collection

In this exploratory study, two semi-structured interview guides (see supplementary materials) were developed based on relevant literature [23, 25, 41] and researcher expertise, addressing topics such as AAS use, interactions with healthcare professionals (specifically pharmacists), and perceptions of incorporating community pharmacy in harm reduction practices. The guides underwent piloting with feedback from the research team and consumers to ensure participant comfort. Specifically, for the AAS guide, this meant collaborative design and review [42] by those with lived experience of AAS, including the first author. These interviews were conducted between December 2022 and April 2023 by a single interviewer (SB), a post-graduate pharmacy student. Similarly, interviews with community pharmacists, led by another pharmacy student (LA) were conducted between July and August 2023, followed a semi-structured format informed by literature [23, 25, 41] and pilot interviews with registered pharmacists (*n* = 2), ensuring minimal changes to the guide. Interviews were conducted via Microsoft Teams and reflective notations were made after each interview, and transcripts were checked for errors, de-identified, and imported into NVivo for analysis.

### Data analysis

A reflexive thematic analysis approach, as described by Braun and Clarke [43], was applied to identify patterns and themes in the data. The analysis was performed by the first author, was iterative, and required multiple rounds of reading and coding the data. Open coding was employed to generate initial codes, followed by focused coding to group related codes, and develop preliminary themes [43]. Regular meetings among the research team were conducted to discuss the emerging codes, themes, and interpretations. This collaborative approach helped ensure a robust analytic procedure [44]. The research team had diverse experiences and backgrounds which was a strength of the research. The first author is a social scientist who has built a professional research relationship within the Australian and international AAS community over the course of well over a decade. He acknowledges his prior lived experience which allowed him to garner the social and body capital to do so [45, 46]. The co-authors, who have extensive experience in community pharmacy research and practice, played a supportive role, offering opportunities for the first author to reflect on the impact of his insider-researcher status on data analysis [47, 48]. To facilitate this reflection, the researchers engaged in reflexive discussions to critically examine their own biases and assumptions and to consider alternative interpretations of the data [49]. The data analysis approach combined inductive and deductive reasoning. Inductive analysis allowed for the discovery of new patterns and themes directly from the data, while deductive analysis ensured alignment with the aims and interview guide [50]. The coding and analytic process persisted until reaching inductive thematic saturation, denoting the point where the accumulated data ceased to offer significant novel insights aligned with the research objectives [51–53]. This analytical approach [23] aimed to capture the richness and depth of participants' perspectives and experiences. The triangulated perspectives are presented below with AAS consumers denoted as ‘Consumers’ and community pharmacists denoted as ‘CPs’.

## Results

### Theme 1: “It would be a really good idea for pharmacists to be aware”: Accessing equipment, information, and overcoming challenges

AAS consumers highlighted the presence of numerous pharmacies in their immediate surroundings, emphasising the ease with which they could access these healthcare destinations. However, there was a disparity in access to AAS injecting equipment between urban and rural areas, fitting with extant work [54]. Consumers in rural areas experienced more pronounced challenges with fewer pharmacies to access. AAS consumers reported instances of limited access to required equipment in specific circumstances, such as being situated in remote rural areas or failing to adequately plan ahead. The contradiction between the participants' proximity to pharmacies and their reported difficulties in accessing equipment suggests a potential discrepancy between availability and actual accessibility.

*Consumer 2*: *It was in the middle of nowhere (ran out of equipment), they (pharmacy) did have an exchange program for the little orange needles for, I guess heroin. So, I know that they had that there, but they didn't have anything in the terms of gauged draw needles for steroids.*

*Consumer 3*: *I've always been able to get what I need. I mean, I'm in [population-dense place], right? Like I got a million pharmacies here. There's always plenty of stuff.*

In triangulating the perspectives of community pharmacists, the data revealed that geographical aspects contributed to their level of knowledge about PIEDs. The geo-spatial aspects of pharmacies posed challenges, particularly if they were situated in areas with limited exposure to PIEDs. Consequently, pharmacists working in these regions had restricted opportunities to encounter PIED users or gain firsthand experience with these substances.

*CP 4: Based on the pharmacy that I work at… out in the country, it's not really that much of an issue.*

*CP 10: I guess the challenge is I would say would be not having enough exposure on how best to provide care and what sort of support resources you can look for.*

In spite of geographical differences, when asked about experiences of not having access to appropriate equipment, AAS consumers indicated that they had, at times, encountered such situations. However, among participants who had experienced ‘running out’ of necessary equipment, a pattern of potentially risky practices emerged, where participants were using far larger needles than required. Alternatively, participants would defer to AAS-using peers to assist them with equipment when possible.

*Consumer 2*: *Yeah, when I was stuck out in the middle of bum steer [‘nowhere’], I didn't really have access to a lot. So, I would have to travel a lot, like a long way, or get a mate to go somewhere and bring it up.*

*Consumer 7*: *Yes and no. Like there’s been once or twice where I've probably run out of barrels or 23-gauge needles, and even in like one circumstance I just used the 19-gauge needle to draw it back and shove it in my glutes. Or I just message a friend and be like “hey bro, do you have any like 25 or three mil barrels?” And they're like, “yeah, man”.*

Obtaining equipment through NSPs, outside pharmacy settings, was common among this cohort. However, participants also identified certain issues associated with NSPs, particularly related to their locations and the stigma associated to substance use outside of AAS specifically. The participants' perspectives on themselves as AAS consumers and their attitudes towards accessing equipment from NSPs revealed concerns and nuanced perceptions. Some participants expressed apprehension about being associated with "more extreme" drug users when obtaining equipment from NSPs.

*Consumer 2*: *Yeah. No, they (NSPs) usually are not great areas. They're usually in back-alley things. Like, I could say I'm not a junkie. Like, I don't do meth(amphetamines), I don't do heroin and that. And that's the kind of clientele these people (NSPs) are having there and it’s like, I don't like kind of associating myself with them. Well, even people that do steroids, I don't really like associating myself with them either, because it's just like. I feel like there's a certain level of ego and, that type of person that comes with it.*

*Consumer 7*: *I'm happy to go to the Community Health Centre, because there is very much, less people, less traffic, you don't have to be so sus about it (obtaining equipment). Like when you go to [place name, NSP], you are kind of like, “oh, I feel like a heroin addict”.*

One participant had a positive experience with NSP workers, particularly with respect to lack of judgement and stigma. However, it could be perceived that a lack of questions were asked when acquiring equipment at NSPs.

*Consumer 6*: *Not a problem at all. I didn't feel that I was being judged. I didn't feel that I was having to be secretive going there or anything like that, because obviously it's like de-identifying. They don't really ask questions.*

While the NSP provided a non-judgmental environment for AAS consumers, there may be potential to foster more proactive engagement, an area which community pharmacists have demonstrated efficacy with other substance consumers [38]. Resultantly, the community pharmacy setting, may offer an alternative approach to consumer care. Some pharmacist’s emphasis on ensuring comprehensive understanding of AAS usage, including injection techniques, adverse effects, and infection risks, highlights the potential for pharmacists to play a crucial role in providing tailored assistance and education to AAS consumers, addressing their specific health concerns and promoting harm reduction practices effectively.

*CP 1: [on service provision with AAS consumer] I wanted to make sure that they’d understand and how to inject… that they understood the adverse effects and potential causes for harm of injecting medications, risk of infection and that sort of thing.*

Parallel with these accounts from pharmacists, consumers reported having predominantly positive experiences engaging with community pharmacies for general health access or AAS-related access. When asked about their previous interactions with pharmacists regarding AAS-related use and their willingness to consult pharmacists in the future for AAS-related concerns, their responses exhibited positive experiences when seeking pharmacist support within the context of their use.

*Consumer 2*: *Once, I had a sore nipple, and I was a bit worried. And then he* (the pharmacist) *was like, “ahh”, because he looked at me, and he was like “are you on anything?”. And I was like, “Yeah”. And then he's like, “yeah, it's a common side effect”. And I was just like, “oh, okay”. I was like “Oh, cheers bro”. And he's like, “yeah, just probably slow it down a bit”.*

When AAS consumers were presented with the hypothetical scenario of the provision of comprehensive AAS ‘kits’ which contained not only necessary equipment, but also harm reduction information from community pharmacies, there was mixed support. Some consumers indicated they would opt to purchase kits from the pharmacy while others saw some limitations which needed to be overcome first, specifically privacy and confidentiality. These types of concerns have been identified in these environments among other substance consumers [9, 12].

*Consumer 2*: *I'd just like to go and buy a kit from the pharmacy, instead of going to the needle exchange.*

*Consumer 6*: *I think the limitation would be standing in line in the checkout, having those kinds of items in your basket. So, I think that maybe in some ways to have it be de-identified.*

Community pharmacists, like the AAS consumers, acknowledged the privacy concerns in these settings [55]. They highlighted the importance of providing a more discreet environment to address these concerns and were receptive to the idea of offering a confidential setting, thus promoting the potential of the space as an enabling resource [33].

*Interviewer: Are there any barriers or challenges that affect the provision of your professional practise or service?*

*CP5: Well, because it's in a community setting and if you've got like two or three other people waiting behind this person…. there's probably other people potentially watching, although I would try and do it in a private setting for this one [AAS use].*

Therefore, amidst these considerations, community pharmacists emerged as potentially receptive to such initiatives. Some pharmacist’s adopted a proactive stance towards assisting AAS consumers, emphasising the importance of maintaining an open and supportive environment within community pharmacies. This attitude suggests a willingness among pharmacists to engage with and accommodate the needs of this specific cohort.

*CP 2: So, that is one of the reasons that I try to be as open and helpful to these patients as I would rather not drive them away from the community pharmacy and our advice.*

*CP 6: I [would] probably broach that subject after some time. If the patients become a regular for us, I'd be comfortable doing that. I'd rather them be safe and take the kit and use it, than not at all.*

In extending on these data, we note AAS consumers specifically emphasised the significance of pharmacists being aware of AAS utilisation and its potential impact on medications being dispensed, reflecting their concern for personal and user group wellbeing. When asked about potential factors that would encourage them to seek AAS-related resources from pharmacies, participants highlighted the importance of increased awareness and information about AAS use.

*Consumer 3*: *I think it would be a really good idea for pharmacists to be aware that people do utilise these substances (AAS) and that potentially is going to impact medications that they're dispensing.*

*Consumer 6: I think for me it would just be awareness [of the pharmacist], which is obviously currently lacking at this point. Like if there was the regular syringes, needles, sharps disposal boxes, alcohol wipes, all the obvious things available at the pharmacy, I'd happily go and get them from there.*

### Theme 2: Nurturing therapeutic alliances: “With that education… I would feel more comfortable in teaching them how to use it”

When AAS consumers reflected on their interactions, affiliations, and perspectives concerning healthcare professionals, a narrative evolved regarding the factors which shape their behaviours and trust towards them. The data substantiate, further, the therapeutic barrier between healthcare professionals and users of AAS [23]. Stigmatisation and negative experiences with healthcare professionals significantly shaped consumers' perceptions of future interactions. Notably, participants' historical backgrounds and relationships with doctors exhibited substantial variation of a therapeutic alliance.

*Consumer 4*: *The other big one would be, if you go and chat to a GP [general practitioner] because you are even at the least bit curious* (about AAS)*. You get a wall put up where they don't wanna chat or converse with you. And they essentially bundle you out of the surgery. You need to then move on to the next one. And like doctor shop, so to speak.*

Community pharmacists also underscored the challenge of establishing open dialogue between AAS consumers. The hesitancy to discuss AAS use creates a barrier to effective communication and potential for rapport-building. These perspectives likely indicate broader systemic barriers faced by AAS consumers in seeking support and guidance from healthcare professionals [56].

*CP 7: The big barrier is probably the [AAS] consumer being hesitant to talk about it. I find that important when you have an open dialogue with someone and in the past, I've had a really good open dialogue with people about different health conditions, whether it's opioid substitution or whether it's. Pseudoephedrine, Clear Eyes [eye drops], or whatever.*

Expanding on this, the longstanding distrust [57] for healthcare professionals appears to have permeated interactions within community pharmacy settings. As a result, pharmacists reported difficulty in engaging with this cohort. There were suggestions to bridge this gap with resources to facilitate discussions and support around AAS use, reflective of work with other healthcare professionals regarding resource creation and dissemination [21, 23]. However, the underlying issue of distrust and reluctance to engage with healthcare professionals, as expressed by AAS consumers, suggests a need for broader systemic changes to foster a more supportive and understanding environment for this population.

*CP 11: I don't think that they are too interested… the ones that I've dealt with that if there was more, maybe patient handouts from the companies or something then at least there's something we could give to the patient. And then maybe sometimes it could make it easier to start conversations and things.*

Building on these perspectives, the main challenge identified by AAS consumers was the difficulty in finding professionals who were receptive to discussing AAS use. Participants expressed a desire to engage in supportive and respectful discussions with healthcare professionals regarding their use, yet they encountered challenges in locating trustworthy contacts who were willing to engage in informed and evidence-based conversations on the topic.

*Consumer 4*: *That's how it kind of works. You gotta know somebody who knows someone, who knows which doctor will be open for conversation as opposed to every doctor.*

Overall, the challenges encountered by consumers in finding healthcare professionals receptive to discussing AAS use highlight the importance of considering broader socio-structural factors that shape drug use behaviours [58]. Within this framework, the concept of community pharmacy as an enabling place [34] emerged as a feasible avenue for accessing professional advice, and participants expressed a preference for services that were convenient and easily accessible. Participants highlighted the nature of the pharmacy experience and its effectiveness in facilitating meaningful encounters.

*Consumer 2*: *They’re very like, something I have noticed about pharmacists, they’re very to the point, which I appreciate in a person, like they’re very clinical as in, “this is a matter of fact. This is the product you want. See you later.”*

Participants were further prompted to express their own perceptions of pharmacies and pharmacists. Responses exhibited a range of viewpoints, reflecting their individual perspectives and attitudes towards these healthcare settings and professionals. These perceptions were shaped by the participants past experiences and interactions, providing valuable insights into the social dynamics and subjective experiences within the community pharmacy context. Overall, pharmacies were noted to be “less confronting” and, potentially, a preferrable site of healthcare provision for this cohort.

*Consumer 3*: *I think pharmacies are less confronting than a medical practitioner.*

*Consumer 2*: *I got a haemorrhoid once from lifting heavy and they’re* (the pharmacist) so good about it. It was just easier instead of going to a doctor and the doctor being like, you know, “let's have a look” *puts glove on*. So, like yeah no I much prefer to go to a pharmacist.

Pharmacist’s perspectives regarding AAS consumers appeared to be centred on reducing harm, specifically there was receptivity towards providing information and education on safer usage of these substances. They emphasised the importance of offering guidance on safety measures and educating consumers. Additionally, they recognised the ‘inevitability’ of substance use and underscored the significance of harm reduction strategies in addressing consumer needs.

*CP 6: You know just providing them with information and education as to how to be safe or safer or just providing some form of guidance as to what to expect. What to look out for, I think is super important. As I said, you know, I think the biggest crux of this thing of these types of drugs is people are going to use it whether you like it or not.*

*CP 11: It's just a field, which I'm not super familiar with. So, it would be good to learn about it and then just see what ways I can help that community.*

Community pharmacists were open regarding the little education they did receive regarding AAS, which reflects current work with healthcare professionals and these substances [21, 25]. The absence of specific training or education on the supply of these drugs indicates a gap in their knowledge, with pharmacists relying primarily on their general training acquired during university studies.

*Interviewer: So, have you ever received or obtained any specific training or education on the supply of performance and image enhancing drugs?*

*CP 9: Not specifically relating those medications. No, it's just, I guess all part of, I guess when you go through uni [university], right, so yeah, nothing more specifically around there, no.*

Collectively, these data underscore a potential need for enhanced education and training programs tailored to equip pharmacists with the knowledge and skills necessary to address the unique needs and concerns of AAS consumers effectively – which community pharmacists are receptive to. Moreover, it highlights the importance of ongoing professional development opportunities to ensure pharmacists remain informed and competent in providing appropriate support and guidance to substance consumers, given they are represented in community pharmacy in a variety of demographics [9, 10, 59–61].

*CP 15: So, I guess with that education we could see what's in there and I would feel more comfortable in teaching them [consumers] how to use it as well as if they have to self-inject.*

## Discussion

This study sought to triangulate the attitudes and experiences of AAS consumers and community pharmacists toward AAS harm reduction within the context of a community pharmacy setting. Notably, our findings underscore the importance of initiating general conversations about AAS use to raise awareness within community pharmacy, an often-overlooked health setting. Raising awareness through public health messaging in other health related areas has demonstrated positive impacts on client attitudes and receptivity in community pharmacy settings [62]. The current data build on extant work demonstrating that AAS consumers express reservations about healthcare professionals providing a safe space for discussions regarding their use of these substances [23, 25, 63]. However, these data also offer promise, given that AAS consumer experiences with community pharmacies are reportedly generally positive. These insights provide a potential avenue for ‘rebuilding’ a lack of trust between AAS consumers and healthcare professionals which has been longstanding [23, 57, 64–66]. Further, given the calls from scholars to look beyond simply dispensing safe-injecting equipment [67, 68], there is an urgent need for further exploration of healthcare provision meeting broader needs of people who use AAS. Our findings provide a way to meet that call and suggest community pharmacies could play a more pivotal role in supporting AAS consumers. This is made more salient given the identified stigma in ‘picking up’ equipment from NSPs which was raised by AAS consumers in the current study. It is, therefore, crucial to consider the social dynamics and contexts that shape consumer perspectives and to foster an ‘enabling place’ [69, 70] which can promote open dialogue and understanding between AAS consumers and healthcare professionals.

### Community pharmacies as an ‘enabling place’ for AAS harm reduction

With the growing availability of AAS through online platforms [71, 72] the traditional reliance on social networks and healthcare providers for access to injecting equipment and safer use information has diminished [73–76]. This unregulated supply of AAS and other PIEDs from online sources is accompanied by misleading information regarding the benefits and risks associated with their use [13, 23], posing significant concerns. Given the potential harms of AAS use among the general population [14, 77], current evidence indicates the potential of their growing impact on the health of this substance cohort globally [78, 79]. Therefore, enhanced harm reduction measures are imperative to effectively engage with the increasing diversity of individuals currently engaged in AAS use [41, 80], particularly considering the potential for emerging dangers associated with the uptake of harsher AAS varieties [81]. Our data indicate that community pharmacies represent sites which can establish an enabling environment conducive to harm reduction for this group, and so we provide an immediate practical application of doing so drawn from our data.

To enhance privacy and confidentiality, community pharmacies can utilise dedicated spaces more effectively, such as private counselling rooms, where AAS consumers can have confidential discussions with pharmacists [55, 82]. Increasing awareness among both pharmacists and consumers about these private spaces has been met with receptivity in relation to mental health [82] and licit substance use [59]. As trust is a crucial component in the pharmacist-consumer relationship, pharmacists have a professional responsibility to establish community pharmacies as ‘safe spaces’ where individuals feel comfortable discussing their health concerns, including AAS use. By adhering to the Code of Ethics, which prioritises the health and wellbeing of consumers [83], pharmacists should set aside judgments and create a non-judgmental environment that fosters open communication. However, our findings revealed a knowledge gap among pharmacists regarding these substances, highlighting the need for further training and education initiatives. Despite this gap, pharmacists demonstrated receptivity to learning and enhancing their understanding of AAS and other PIEDs, indicating a potential for improved engagement in harm reduction efforts within community pharmacy settings. Further research is needed to understand knowledge gaps, training needs, and the effectiveness of educational interventions for pharmacists in addressing AAS use. These efforts can contribute to fostering a conducive environment for harm reduction for AAS consumers, a necessity that demands immediate attention.

### Limitations

This study represents a first known attempt at exploring the relationship between AAS consumers and community pharmacy, however is not without limitations. Recruitment via personal and professional networks may have introduced social desirability bias, potentially impacting the credibility of participant responses. It is important to acknowledge it is possible that our findings may not fully capture the complete range of experiences by AAS consumers or community pharmacists. Lastly, the study findings should be interpreted within the context of Australian practice, and it is important to acknowledge that variations may exist in different study contexts.

### Conclusions

Overall, the findings underscore the continued importance of establishing therapeutic alliances between AAS consumers within the community pharmacy setting. There is a clear need for increased awareness and information about AAS use among pharmacists and other healthcare professionals. Further research is needed to close the knowledge gap among community pharmacists regarding AAS-specific education, enabling the delivery of more comprehensive harm reduction. However, fostering non-judgmental and supportive interactions should be considered the first step to contributing to safer AAS use practices and better healthcare outcomes for AAS consumers.

### Supplementary Information

**Additional file 1: Supplementary Materials.**

## Data Availability

De-identified data may be available from the team on reasonable request.
