# Supplementary material for: Exploring the role of community pharmacies as a harm reduction environment for anabolic–androgenic steroid consumers: triangulating the perspectives of consumers and pharmacists
Source: Harm Reduct J. 2024 Mar 13;21:59. doi: 10.1186/s12954-024-00972-5 (PMC10935940; doi:10.1186/s12954-024-00972-5)
Supplement: Supplementary file 1 — Additional file 1: Supplementary Materials. [file 12954_2024_972_MOESM1_ESM.docx]

**Supplementary Materials – Appendices**

**Appendix A**

**Semi-structured Interview Guide**

1. Informed consent /plain language statement process

Have you read the Patient Consent form

1. Information regarding participant

Prompts include

- Age
- Gender
- Work/Study

1. Could you tell me more about your exercise background?
   1. Do you use any health supplements and, if so, what kind?
   2. What would you say drives you to want to be muscular/fit?
   3. Do you have an end goal? If so, what is it?
2. What encouraged you to start using PIEDs?
   1. How do you feel since using PIEDs - Could you tell me a little bit about your experience?
3. What do you know about safe injecting practice?
   1. Do you have any concerns when it comes to injecting?
   2. Who do you go to for advice about injecting?
4. What does harm minimisation mean to you?
   1. Have you ever accessed a harm minimisation service before (like a needle exchange)?
   2. If so, could you tell me a little bit about your experience?
5. Do you have access to all the necessary equipment for using PIEDs?
   1. What is your necessary equipment?
   2. Have you ever been in a situation where you had no access to supplies?
   3. If so, what did you do?
   4. Have you ever shared any injecting equipment? If so could you tell me about that?
   5. Have you ever shared any vials? If so could you tell me about that?
   6. Have you ever had any issues with infections or abscesses? If so could you tell me about that?
6. How often do you visit a pharmacy for any service/product?
   1. Do you have a regular pharmacy you access?
7. Have you ever been to a pharmacy for reasons related to your PIED use?
   1. If so, why, if not, why not?
   2. Have you ever received harm minimisation information from a pharmacist before?
   3. What support tools do you know of that pharmacies have available to the public for any injecting related practices?
   4. Have you ever had a negative interaction with a pharmacist/pharmacy staff before? If so, could you tell me a little bit about that?
   5. Have you ever had a positive interaction with a pharmacist/pharmacy staff before? If so, could you tell me a little bit about that?
8. What would you like to be made available to you within a pharmacy setting related to PIED use?

Do you want anymore information? Or to add anything?

Would you like a copy of the transcript from this interview?

**Prompts added overtime**

- Has a health practitioner/GP ever prompted you to go to the pharmacy for PIED related reasons?
- If a doctor or another health professional you confide in, prompted you to seek information and advice from a pharmacist, would you?
- If kits were made available within pharmacy for purchase, would you be inclined to buy them?

**Appendix A**

Semi-structured Interview Guide (for community pharmacists)

1. Informed consent /plain language statement process
2. Information regarding participant

*Prompts include (if workforce):*

*- Age*

*- Gender*

*- Participant’s working background*

*- Number of years working*

*- Any other important information*

1. Please describe how you currently interact with PIED consumers?
2. How confident are you in terms of the equipment which PIED users require from your service? (Why / why not?)
3. Could you tell me a bit more about what consumers using PIED seek from you in terms of service/s?
   1. Could you give me an example?
4. What about other drug consumers?
5. How confident are you with managing infections and BBVs related to PIEDs?

- What about STIs?

1. Do you believe there is room for any harm reduction work to be done with PIED consumers in the community pharmacy setting? If so, what? Barriers/Facilitators to this?
2. Would you be confident to provide safe injecting advice or information about reducing harms to PIED users? Why/Why not?

Conclusion of interview

*Prompts include:*

*- Any other issues participant wants to raise that were not covered in the interview?*

*- Does participant want a transcript of this interview? Summary of research findings?*

**Appendix C.**

### COREQ Criteria Checklist

**Developed from:**

Tong A, Sainsbury P, Craig J. Consolidated criteria for reporting qualitative research (COREQ): a 32-item checklist for interviews and focus groups. *International Journal for Quality in Health Care*. 2007. Volume 19, Number 6: pp. 349 – 357

| **No.** | **Item** | **Guide Questions/Description** | **Notes and/or Section Reported in** |
| --- | --- | --- | --- |
| **Domain 1: Research team and reﬂexivity** | | | |
| *Personal Characteristics* | | | |
| 1 | Interviewer/  facilitator | Which author/s conducted the interview or focus group? | Sarah Benn and Lkhagvadulam Ayurzana |
| 2 | Credentials | What were the researcher’s credentials? E.g. PhD, MD | The interviewers were both Master of Pharmacy Students. All other members of the research team have PhD’s. |
| 3 | Occupation | What was their occupation at the time of the study? | The interviewers were students and were employed in community pharmacy part-time. All other members of the research team are academics. |
| 4 | Gender | Was the researcher male or female? | Sarah Benn (the interviewer) and A/Prof Hattingh, A/Prof King, and Dr McMillan are female, Dr Piatkowski is male. |
| 5 | Experience and training | What experience or training did the researcher have? | The interviewers were Master of Pharmacy students who had been employed part-time in a community pharmacy for one year at the time of the study. All other members of the research team have doctorate level qualifications and a long history in qualitative research. Dr Piatkowski is also a peer researcher with extensive research in co-designing research with people who use drugs. |
| *Relationship with participants* | | | |
| 6 | Relationship established | Was a relationship established prior to study commencement? | No. Initial contact was only to establish interest in the study by providing the Participant Information Sheet and to organise a time for interview. |
| 7 | Participant knowledge of the interviewer | What did the participants know about the researcher? e.g. personal goals, reasons for doing the research | Only information provided in the Participant Information Sheet and Interview Guide. See Method section and Interview Guide. |
| 8 | Interviewer characteristics | What characteristics were reported about the interviewer/facilitator? e.g. Bias, assumptions, reasons and interests in the research topic | Only information provided in the Participant Information Sheet and Interview Guide. See Method section and Interview Guide. |
| **Domain 2: Study design** | | | |
| *Theoretical framework* | | | |
| 9 | Methodological orientation, ontological or epistemological basis | What methodological orientation was stated to underpin the study? e.g. grounded theory, discourse analysis, ethnography, phenomenology, content analysis | Exploratory in nature. We utilised an ontological lens grounded in sociological theory as outlined in our framing. Methodologically, this was paired with reflexive TA. See Method section for more information. |
| *Participant selection* | | | |
| 10 | Sampling | How were participants selected? e.g. purposive, convenience, consecutive, snowball | Purposive and snowball sampling. See Method section for more information. |
| 11 | Method of approach | How were participants approached? e.g. face-to-face, telephone, mail, email | Described in the Method section. |
| 12 | Sample size | How many participants were in the study? | A total of 23 participants: 8 AAS consumers and 15 community pharmacists agreed to participate in this study. |
| 13 | Non-participation | How many people refused to participate or dropped out? Reasons? | 23 interviews were conducted. See Methods section for more information. |
| 14 | Setting of data collection | Where was the data collected? e.g. home, clinic, workplace | Collection of data was via the recording of a Microsoft Teams interview with participants. The researcher recommended participants to be a in quiet place free from distractions during the duration of the interview. |
| 15 | Presence of non-participants | Was anyone else present besides the participants and researchers? | Unable to be determined as interviews were conducted over Teams. No other persons were present on the researcher’s end. |
| 16 | Description of sample | What are the important characteristics of the sample? e.g. demographic data, date | Characteristics of the samples are reported in the Methods section. |
| *Data collection* | | | |
| 17 | Interview guide | Were questions, prompts, guides provided by the authors? Was it pilot tested? | Refer to Interview Guide (Appendix A and B). The Interview Guide was pilot tested through the use of mock interviews with members of the research team and members of the AAS-using community. For Interview guide A particularly, there was a substantive co-design process employed. Adjustments were made to the Interview Guide after the first participant interview. See Method section for more information. |
| 18 | Repeat interviews | Were repeat interviews carried out? If yes, how many? | No repeat interviews were conducted with participants. |
| 19 | Audio/visual recording | Did the research use audio or visual recording to collect the data? | Audio recording only. See Method section for more information. |
| 20 | Field notes | Were ﬁeld notes made during and/or after the interview or focus group? | Field notes were taken during and after the interviews. See Method section for more information. |
| 21 | Duration | What was the duration of the interviews or focus group? | 30-60-minute interviews. |
| 22 | Data saturation | Was data saturation discussed? | Yes. See Method section. |
| 23 | Transcripts returned | Were transcripts returned to participants for comment and/or correction? | Transcripts were offered to participants for review. Two community pharmacy participants took up the offer. |
| **Domain 3: Analysis and findings** | | | |
| *Data analysis* | | | |
| 24 | Number of data coders | How many data coders coded the data? | One data coder coded the data in collaboration with the whole research team. |
| 25 | Description of the coding tree | Did authors provide a description of the coding tree? | Yes. See Method section. |
| 26 | Derivation of themes | Were themes identiﬁed in advance or derived from the data? | Themes were derived from the data. See Method section. |
| 27 | Software | What software, if applicable, was used to manage the data? | Qualitative data analysis software NVivo Version 12 was used to organise the interview transcripts. |
| 28 | Participant checking | Did participants provide feedback on the ﬁndings? | No, but copies of the findings were provided to all participants. |
| *Reporting* | | | |
| 29 | Quotations presented | Were participant quotations presented to illustrate the themes/ﬁndings? Was each quotation identiﬁed? e.g. participant number | Yes. See Findings section. |
| 30 | Data and ﬁndings consistent | Was there consistency between the data presented and the ﬁndings? | Yes, findings were directly derived from the data. See Findings and Discussion sections for more information. |
| 31 | Clarity of major themes | Were major themes clearly presented in the ﬁndings? | Yes. Themes were discussed in the Findings section. See Findings section for more information. |
| 32 | Clarity of minor themes | Is there a description of diverse cases or discussion of minor themes? | Data was highly homogenous. Minor discussion of diverse cases occurs in the Findings section. |
